# Gastrointestinal and Peritoneal Barium Granulomas in Old Patient

**DOI:** 10.5334/jbsr.2063

**Published:** 2020-04-07

**Authors:** Osamah Alwalid, XueHua Shen

**Affiliations:** 1 Union Hospital, Tongji Medical College, Huazhong, University of Science and Technology, Wuhan, CN

**Keywords:** Barium Sulfate, Barium Granuloma, Barium Stone

## Abstract

**Teaching point:** Barium granuloma is a rare but potentially risky complication of barium studies that should be prevented, especially in susceptible patients.

## Case History

**Figure 1:**
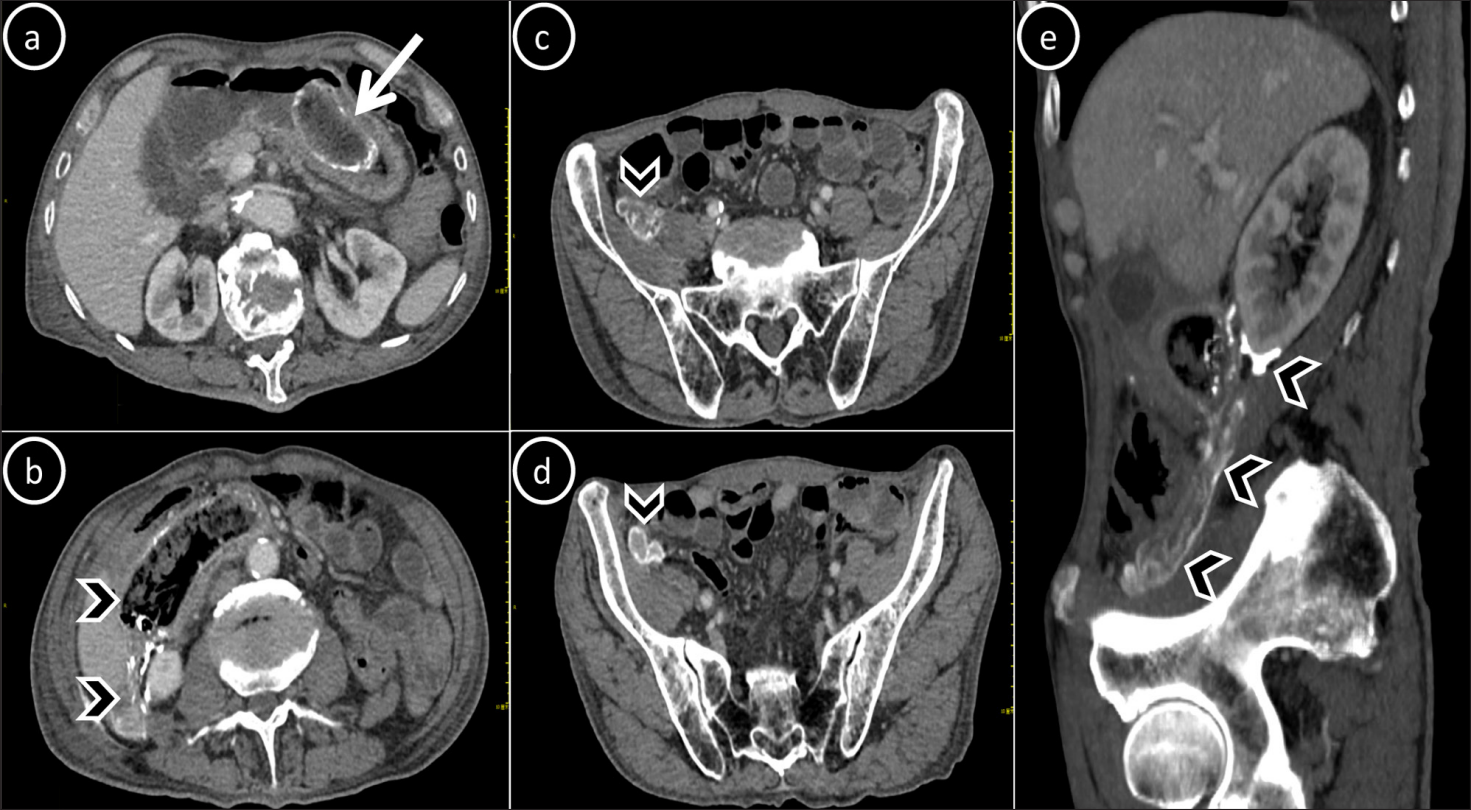
Axial **(a–d)** and sagittal reconstructed **(e)** contrast-enhanced abdominal CT scan of an 81-year-old patient with gastrointestinal (arrow) and peritoneal (arrowheads) barium granulomas.

An 81-year-old man presented with a two-month history of abdominal distention and pain with on-and-off fevers. There was a clinical suspicion of intestinal obstruction and a subsequent CT scan was performed. Abdominal CT (Figure 1) revealed two large structures; one of which was located within the gastric lumen (Figure 1a, arrow), and the other one was abutting the third part of the duodenum and extending posteriorly into the pararenal space and along the right paracolic gutter into the right iliac fossa (Figure 1b–e, arrowheads) with surrounding peritoneal fat stranding and fluid collection. Both structures featured calcified shell and heterogeneous hypodense contents and gas. Signs of incomplete intestinal obstruction were also present. Further questioning of the patient revealed a history of upper gastrointestinal barium study. Based on the patient’s history and typical imaging findings, the diagnosis of barium granuloma formation complicated by intestinal obstruction, perforation and subsequent peritonitis was given. Unfortunately, because of the old age and other co-morbidities, the patient wasn’t eligible for surgical intervention and passed away a few days after discharge.

## Comment

Barium granuloma, also called barytoma or barium stone formation, is a rare complication of the barium contrast use in the gastrointestinal tract. It has been mostly described in the colon after barium enema studies but may occur in any part of the alimentary tract. It is often an incidental finding; however, it may rarely produce a polypoid, ulcerated or intramural lesion resembling a neoplasm. Barytoma may erode the mucosa and cause perforation, in which case it may remain visible for years or may result in acute chemical or chronic granulomatous peritonitis [1]. Pre-existing ulceration and Parkinson’s disease were reported as risk factors. In our case, diminished bowel motion and delayed gastric emptying in old age could have predisposed to the barium retention and stone formation. Other risk factors encountered in this condition include bowel inflammation, and mucosal injuries post instrumentations [1]. Therefore, history acquisition, meticulous procedure and immediate evacuation of the barium from the gastrointestinal tract, especially in susceptible patients, are essential to prevent this complication.

## References

[1] de Feiter PW, Soeters PB, Dejong CHC. Rectal Perforations After Barium Enema: A Review. Diseases of the Colon & Rectum. 2006 2 01; 49(2): 261–71. DOI: 10.1007/s10350-005-0225-3 16328608

